# The Role of the Subthalamic Nucleus in L-DOPA Induced Dyskinesia in 6-Hydroxydopamine Lesioned Rats

**DOI:** 10.1371/journal.pone.0042652

**Published:** 2012-08-06

**Authors:** Asier Aristieta, Garikoitz Azkona, Ainhoa Sagarduy, Cristina Miguelez, José Ángel Ruiz-Ortega, Rosario Sanchez-Pernaute, Luisa Ugedo

**Affiliations:** 1 Department of Pharmacology, Faculty of Medicine and Dentistry, University of the Basque Country, Leioa, Spain; 2 Laboratory of Stem Cells and Neural Repair, Fundacion Inbiomed, San Sebastian, Spain; National Institutes of Health, United States of America

## Abstract

L-DOPA is the most effective treatment for Parkinson's disease (PD), but prolonged use leads to disabling motor complications including dyskinesia. Strong evidence supports a role of the subthalamic nucleus (STN) in the pathophysiology of PD whereas its role in dyskinesia is a matter of controversy. Here, we investigated the involvement of STN in dyskinesia, using single-unit extracellular recording, behavioural and molecular approaches in hemi-parkinsonian rats rendered dyskinetic by chronic L-DOPA administration. Our results show that chronic L-DOPA treatment does not modify the abnormal STN activity induced by the 6-hydroxydopamine lesion of the nigrostriatal pathway in this model. Likewise, we observed a loss of STN responsiveness to a single L-DOPA dose both in lesioned and sham animals that received daily L-DOPA treatment. We did not find any correlation between the abnormal involuntary movement (AIM) scores and the electrophysiological parameters of STN neurons recorded 24 h or 20–120 min after the last L-DOPA injection, except for the axial subscores. Nonetheless, unilateral chemical ablation of the STN with ibotenic acid resulted in a reduction in global AIM scores and peak-severity of dyskinesia. In addition, STN lesion decreased the anti-dyskinetogenic effect of buspirone in a reciprocal manner. Striatal protein expression was altered in dyskinetic animals with increases in ΔFosB, phosphoDARPP-32, dopamine receptor (DR) D3 and DRD2/DRD1 ratio. The STN lesion attenuated the striatal molecular changes and normalized the DRD2/DRD1 ratio. Taken together, our results show that the STN plays a role, if modest, in the physiopathology of dyskinesias.

## Introduction

Parkinson disease (PD) involves the degeneration of dopamine (DA) neurons in the substantia nigra (SN) and a subsequent DA depletion in the striatum [1]. Pharmacological replacement with L-3,4-dihydroxyphenylalanine (L-DOPA) is the gold standard treatment for PD. However, long-term administration of L-DOPA induces abnormal involuntary movements known as L-DOPA-induced dyskinesia (LID) in the majority of PD patients. These motor complications are potentially disabling, and affect up to 40% of PD patients within 5 years of treatment [2]. Although the specific mechanisms underlying LID are poorly understood there is vast consensus that it results from dysregulated DA neurotransmission depending on both presynaptic alterations and post-synaptic dopamine receptor (DR) supersensitivity [3]. Altered glutamatergic transmission within the basal ganglia may also be involved since changes in NMDA and metabotropic glutamate receptors (mGluR) have been reported.

The basal ganglia form a highly organized network and the subthalamic nucleus (STN) occupies a prominent position in the indirect pathway [4], [5]. According to the classical basal ganglia model [6] the loss of DA in the parkinsonian condition leads to a disinhibition of the indirect pathway -i.e. the lack of DA, which is inhibitory on DRD2-bearing medium spiny neurons projecting to the external globus pallidus (GPe), decreases the GPe inhibitory input on the STN resulting in an activation of STN-. In addition to a dysregulation of DA acting directly on basal ganglia output structures, and the above-mentioned decrease in GPe inhibition, direct cortical and thalamic excitatory projections can also influence STN activity [7]. In any case, the increase in STN activity has been extensively documented in patients and experimental models of PD [8]–[12]. In contrast, there are few studies showing the effect of prolonged L-DOPA administration on STN activity and the impact of STN activity on LID. Most results obtained in patients undergoing subthalamic stimulation conclude that the reduction of dyskinesias is due to the reduction of L-DOPA administration [13]. On the other hand, a few studies suggest that STN stimulation *per se* has an antidyskinetic effect [14]–[16] while still others claim it exacerbates dyskinesia [17], [18].

The present study aimed at defining the role of the STN in dyskinesia and corresponding molecular changes in the striatum, induced by prolonged treatment with L-DOPA in 6-hydroxydopamine (6-OHDA)- lesioned rats. Our results demonstrate that the lesion of the STN reduces the LID and partially normalizes the aberrant striatal molecular changes associated with LID. Taken together, this study shows that the STN is involved in the physiopathology of dyskinesia.

## Materials and Methods

### Animals

Sprague-Dawley rats (Harlan) weighing 200–250 g at the beginning of the experiments were housed in groups of five in standard laboratory conditions (22±1°C, 55±5% of relative humidity, and a 12∶12 h light/dark cycle) with *ad libitum* access to food and water. Every effort was made to minimize animal suffering and to use the minimum number of animals per group and experiment. Experimental procedures were approved by the Local Ethical Committee of the University of Basque Country (UPV/EHU), following the Communities Council Directive 2010/63/UE and Spanish (RD 1201/2005) regulations for the care and use of laboratory animals.

### Drugs

The following drugs were used in this study: 6-hydroxydopamine (6-OHDA; 3.5 µg/µl), desipramine hydrochloride (25 mg/kg i.p.), pargyline (50 mg/kg i.p.), amphetamine sulfate (3 mg/kg i.p.), L-DOPA (L-3,4-dihydroxyphenylalanine methyl ester hydrochloride, 6 mg/kg i.p.), benserazide-HCL (12 mg/kg i.p.), ibotenic acid (α-amino-3-hydroxy-5-isoxazoleacetic acid; 10 µg/µl) and buspirone (4 mg/kg, i.p.) from Sigma-Aldrich. Desipramine, pargyline, L-DOPA, benserazide and amphetamine sulfate were prepared in 0.9% saline. 6-OHDA was dissolved in MiliQ water containing 0.02% ascorbic acid. Ibotenic acid was dissolved in Dulbecco's buffered saline solution containing (in mM): NaCl 136.9, KCl 2.7, NaH_2_PO_4_ 8.1, KH_2_PO4 1.5, MgCl_2_ 0.5 and CaCl_2_ 0.9 (pH = 7.40). All drugs were prepared on the day of the experiment.

### Experimental design

**Figure 1:**
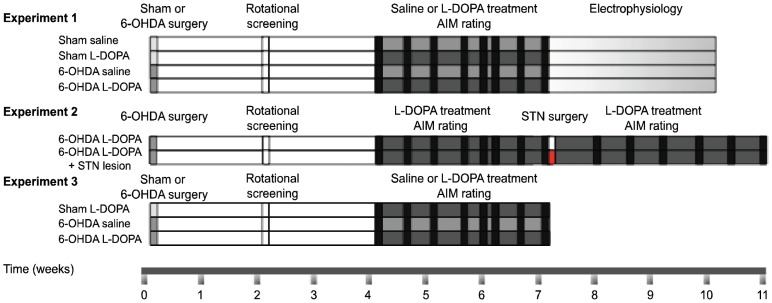
Schematic representation of experimental design and groups. At the beginning of the study, animals received a vehicle or 6-OHDA injection into the right medial forebrain bundle and were screened by amphetamine-induced rotations 2 weeks later. Rats were treated daily with saline or L-DOPA injections (6 mg/kg plus 12 mg/kg benserazide) for 21 days. AIMs were rated 2–3 days per week (testing sessions are marked in black). ***Experiment 1***: After the chronic treatment was completed, L-DOPA was administered twice per week, and electrophysiological experiments were performed. All animals were perfused transcardially and processed for histology. ***Experiment 2***: After the last testing session (day 21), the animals received a vehicle (in white) or ibotenic acid (in red) injection into the right STN. After a second period of L-DOPA treatment and AIMs rating (in black) animals were killed and the striata were dissected and frozen for western blot, and STN was postfixed for histology. ***Experiment 3***: After chronic treatment and AIMs rating (in black), animals were killed and striata removed and frozen for western blot analysis.

The design and timeline of the experiments is shown in **Figure 1**. **Experiment 1**. Characterization of the electrophysiological properties of STN neurons in sham and 6-OHDA-lesioned animals treated chronically (3 weeks) with either saline or L-DOPA. Behavioral tests were performed periodically. All the 6-OHDA-lesioned animals treated with L-DOPA included in these experiments developed dyskinesia. Electrophysiological recordings were obtained 24 h after the last dose of the corresponding treatment (saline or L-DOPA). After baseline recordings were acquired, rats received an acute challenge with a standard dose of L-DOPA (6 mg/kg plus benserazide 12 mg/kg, i.p.) and cell recordings were obtained from 20 to 120 min after the drug administration. The groups included in this experiment are referred to as sham saline (n = 9), sham L-DOPA (n = 12), 6-OHDA saline (n = 12) and 6-OHDA L-DOPA (n = 13). **Experiment 2**. Impact of STN lesion on LID. Two groups of dyskinetic animals received an injection in the STN with ibotenic acid (STN-lesion; n = 14) or Dulbeco's buffer solution (STN-sham; n = 5). Abnormal involuntary movement (AIM) scores were evaluated before and after the STN surgery. At the end of the experiment, 6 rats from the STN-lesion and 5 from the STN-sham group received a single buspirone injection 30 min prior to L-DOPA administration. **Experiment 3**. Effect of STN lesion on striatal protein expression. For this purpose, animals were killed 1 h after the last saline or L-DOPA injection and the striata were processed for western blot analyses. The groups included in this experiment are referred to as sham L-DOPA (n = 7), 6-OHDA saline (n = 9), 6-OHDA L-DOPA (n = 10) and STN lesion (n = 8 from Experiment 2. Animals tested with buspirone were excluded from this analysis).

### 6-OHDA lesion and rotational screening

Thirty minutes before the surgery, rats were pre-treated with desipramine in order to protect noradrenergic terminals from 6-OHDA toxicity, and with pargyline to inhibit monoamine oxidase activity. Rats were deeply anesthetized with isoflurane (1.5–2%, Esteve) and 6-OHDA or vehicle was infused (1 µl/min) using a Hamilton microsyringe. A total of 15.75 µg in 4.5 µl were injected in two sites at right medial forebrain bundle: 2.5 µl at anteroposterior (AP) −4.4 mm, mediolateral (ML) +1.2 mm, and dorsoventral (DV) −7.8 mm, relative to bregma and dura with the toothbar set at −2.4, and 2 µl at AP −4.0 mm, ML +0.8 mm, and DV −8 mm, with the toothbar at +3.4 [19].

The turning behaviour was recorded 2 weeks post-surgery over 90 minutes after amphetamine sulfate administration as indicator of DA denervation [20]. Animals were selected for the study and randomly assigned to one of the 6-OHDA groups.

### Abnormal involuntary movement rating

Abnormal involuntary movements (AIMs) were induced in 6-OHDA-lesioned rats by chronic daily injections of L-DOPA (6 mg/kg, i.p) in combination with the peripheral decarboxylase inhibitor benserazide (12 mg/kg, i.p.) over 3 weeks. AIMs were scored according to a rat dyskinesia scale described in [21]. Once the scores reached a plateau, L-DOPA dosing was changed to a maintenance (twice a week) schedule [22]. On the testing days, rats were placed individually in transparent empty plastic cages for at least 10 min prior to drug administration. Following L-DOPA injection, each rat was observed for one full minute every 30^th^ (experiment 1) or 20^th^ (experiment 2 and 3) min, for a total of 180 min (experiment 1) or 240 min (experiment 2 and 3). The severity of the three subtypes of dyskinetic movements (axial, limb and orolingual) AIMs and asymmetric locomotive behaviour (locomotive AIMs) were rated from 0 to 4, based on the amount of time in which the abnormal movement was present during the observation period (i.e. 0, not present to 4, continuous). In addition, the amplitude of axial, limb and orolingual AIMs was rated on a scale from 0 to 4. Axial, limb, and orolingual AIMs were analyzed separately from locomotive AIMs. The AIM score/session for axial, limb and orolingual AIMs is the result of multiplying the severity by the amplitude scores for each monitoring period, with all these products summed for each testing sessions [23]. Locomotive AIMs score/session rates only the severity of the abnormal movement.

### Electrophysiological procedures

The electrophysiological characteristics of STN neurons were investigated after 3 weeks of saline or L-DOPA treatment as described in detail in [24]. Briefly, rats were anesthetized with urethane (1.2 g/kg, i.p.) and placed in a stereotaxic frame with its head secured in a horizontal orientation. The recording electrode, consisting of an Omegadot single glass micropipette with a tip diameter of 1–2.5 µm and filled with a 2% solution of Pontamine Sky Blue in 0.5% sodium acetate was lowered into the right STN (relative to bregma and dura: AP −3.6–3.8 mm, ML +2.2–2.7 mm and DV −7–8 mm [19]).The extracellular signal from the electrode was amplified with a high-input impedance amplifier, and then monitored on an oscilloscope and on an audio monitor. All recorded STN neurons exhibited a biphasic waveform with a duration of 1.0 to 1.5 ms as previously described [25]. Neuronal spikes were digitized using computer software (CED micro 1401 interface and Spike2 software, Cambridge Electronic Design). The basal firing rate was recorded for 5–10 min. At the end of experiments, a 5 µA cathodal current was passed through the recording electrode, thus depositing a discrete spot of Pontamine Sky Blue to mark the recording site.

The firing parameters of STN neurons were analyzed off-line using the Spike2 software. The following parameters were calculated: firing rate and coefficient of variation, which is a measure of firing regularity and is defined as the percentage ratio of the standard deviation to the mean interval histogram. According to the method of [26], based on the concept of density distribution and on a statistically rigorous definition of the firing pattern, three different firing patterns could be analyzed (using the NeuronFit program from NorayBio Informatics): (1) a tonic firing pattern characterized by a symmetrical density distribution histogram, (2) a random firing pattern characterized by a Poisson distribution and (3) a bursting firing pattern characterized by a distribution histogram which is significantly different (*p*<0.05) to a Poisson distribution, presenting a significantly positive skewness (*p*<0.05) of the density distribution histogram and a minimum of 4 spikes per burst. The analyzed spike-trains lasted more than 120 s and contained at least 300 spikes.

### Subthalamic nucleus lesion

On day 22^nd^ from the beginning of L-DOPA chronic treatment, the right STN was lesioned as previously described [27]. Animals were anesthetized with isoflurane (1.5–2%, Esteve) and 1 µl of ibotenic acid or Dulbecco's buffer solution was infused (0.5 µl/min) into the right STN at AP −3.8 mm, ML +2.5 mm, and DV −8.0 mm, relative to bregma and dura [19], using a micropipette connected to a Hamilton syringe. Five days after the STN lesion, AIMs were evaluated for another 27 days in 6 additional sessions.

### Histological procedures and analysis

Immediately after the electrophysiological procedure animals were deeply anaesthetized and transcardially perfused with saline followed by 4% ice-cold paraformaldehyde, prepared in 0.1 M phosphate buffer. Brains were removed and transferred to a 25% sucrose solution until they sank. The brains were serially cut in coronal 40 µm sections using a freezing microtome (HM 430, Microm), and slices were conserved in a cryoprotective solution at −20°C until further processing.

Tyrosine hydroxylase (TH)-immunostaining was used to examine the degree of DA denervation in the striatum and the substantia nigra. Briefly, after endogenous peroxidases inactivation, sections were blocked with 5% normal goat serum, and incubated with rabbit anti-TH (AB 152, 1∶1000, Chemicon) primary antibody for 36 h at 4°C. The sections were then incubated for 2 h with a biotinylated goat antibody against rabbit IgG (BA 1000, 1∶200, Vector Laboratories). Thereafter, sections were incubated with an avidin–biotin–peroxidase complex (ABC kit, PK-6100, Vector Laboratories) and peroxidase activity was visualized with 0.05% 3,3′-diaminobenzidine (Sigma) and 0.03% hydrogen peroxide. Finally, sections were mounted onto gelatin-coated slides, dehydrated, cleared with xylene and coverslipped. For the analysis, three striatal sections for each animal (rostral, medial, and caudal levels) were optically digitized and the mean optical density (OD) associated with the striatum was calculated using NIH-produced image analysis software [ImageJ (http://rsb.info.nih.gov/nih.image/default.html)]. The OD was expressed as a percentage of that of the contralateral intact side (100%) with the background associated with the cortex being set as 0%.

To verify the ibotenic acid lesion thionine staining was performed on brain sections containing the STN as described [27]. Neuronal loss was evaluated using a light microscope (Nikon Optiphot, ×10 objective) and stereological cell counting was performed in 4 regularly spaced sections along the STN by the optical dissector method using the Mercator image analysis system (Explora Nova) as described in [20].

For the location of the recording site, brain sections containing the STN were mounted on gelatinized glass slides, stained with 1% neutral red, washed, dehydrated, cleared with xylene and coverslipped. Only cells recorded within the STN have been included in the study.

### Western blot

One hour after the last saline or L-DOPA injection animals were killed by decapitation. The brains were then removed, right and left striatum were dissected on an ice-cold surface and quickly frozen. For protein extraction [28] each striatum was homogenized in ice-cold lysis buffer (10 mM HEPES pH 7.5, 150 mM NaCl, 1 mM EDTA, 0.1 mM MgCl_2_, phosphate-buffered saline (PBS) 0.2% Triton X-100) and a protease inhibitor cocktail (Roche). After clearance of the lysates by centrifugation (16000×g, 30 min at 4°C), protein quantification was performed following the DC Protein Assay (Bio-Rad Laboratories) protocol. Equal amount of protein (50 µg) for each sample was then separated in SDS- polyacrylamide gels (SDS-PAGE) and subsequently transferred to polyvinylidene difluoride (PVDF, Millipore) membranes. Blots were blocked with 5% non-fat dry milk or bovine serum albumine Tris-buffered saline including 0.1% Tween-20 (TBS-T) and incubated overnight at 4°C with the following primary antibodies: mouse anti-TH (1∶1000; Millipore) and anti-DRD3 (1∶1000, Santa Cruz Biotechnology), rabbit anti-cFos (1∶1000; Santa Cruz Biotechnology), anti-FosB (1∶200; Santa Cruz Biotechnology), anti-mGluR5 (1∶1000; Novus Biologicals), anti-DRD1 (1∶1000; Sigma-Aldrich), anti-DRD2 (1∶1000; Lifespan Biosciences), anti-pDARPP32 (Thr34) (1∶1000; Cell Signaling Technology), anti-DARPP32 (1∶1000; Cell Signaling Technology), anti-Actin (1∶1000; Sigma-Aldrich), and anti-GAPDH (V-18) HRP (1∶1000; Santa Cruz Biotechnology). Incubation with horseradish peroxidase (HRP)-conjugated anti-mouse or anti-rabbit IgG (GE Healthcare), followed by enhanced chemiluminescence (Super Signal, Thermo Scientific) detection system. Quantification was made by densitometric analysis of non-saturated films using Image J software.

### Statistical analysis of data

Experimental data were analyzed using the computer program GraphPad Prism (v. 5.01, GraphPad Software, Inc). TH OD in the striatum and thionine cell counts in the STN were analyzed using paired t test (lesioned versus unlesioned side). The electrophysiological data were analyzed by one-way analysis of variance (ANOVA) followed by *post-hoc* comparisons using the Bonferroni test. The firing pattern of STN neurons was analyzed using Fisher's exact test. The effect of STN lesion on AIM scores was evaluated using two-way repeated measures (RM) ANOVA when STN-lesion and STN-sham were compared and one-way RM ANOVA when pre- and post-injection scores were compared in each group. Western blot data were normalized to loading control and expressed as right to left ratios; group comparisons were performed using one-way ANOVA followed by Bonferroni *post-hoc* tests. Simple linear regression analyses were used to assess correlations between different data. The level of statistical significance was set at *p*<0.05. Data are presented as group means ± standard error of the mean (S.E.M.).

## Results

### L-DOPA-induced abnormal involuntary movements in hemiparkinsonian rats

**Figure 2:**
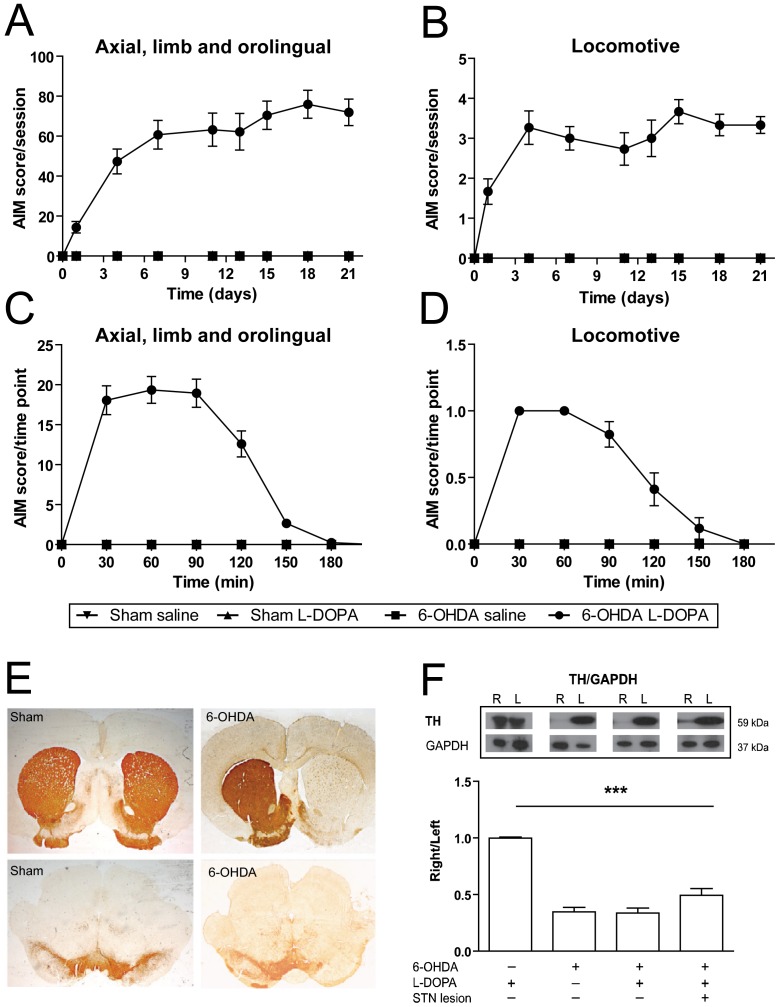
Characterization of hemiparkinsonian rats with L-DOPA induced dyskinesia. Evolution of dykinesia scores showing *(* ***A*** *)* the sum of AIM scores for axial, limb and orolingual ratings and *(* ***B*** *)* locomotive score, during L-DOPA chronic treatment. Time course of *(* ***C*** *)* axial, limb and orolingual AIMs score and *(* ***D*** *)* locomotive scores evaluated after a single injection of L-DOPA on the last testing session (day 21st). Groups: sham saline (n = 9), sham L-DOPA (n = 12), 6-OHDA saline (n = 12) and 6-OHDA L-DOPA (n = 13). Note that animals in the sham saline, sham L-DOPA and 6-OHDA saline groups did not develop any AIMs. *(* ***E*** *)* Photomicrographs showing the extensive loss of TH positive fibers in the striatum (upper right panel) and TH positive cells in the substantia nigra (lower right panel) ipsilateral to 6-OHDA lesion. All 6-OHDA lesioned animals included in this experiment showed >95% reduction in TH-fiber optical density in the lesioned striatum. Groups: sham saline (n = 9), sham L-DOPA (n = 12), 6-OHDA saline (n = 12) and 6-OHDA L-DOPA (n = 13). *(* ***F*** *)* Western blot results showing a significant reduction of TH expression in all the 6-OHDA lesioned groups. Groups: sham L-DOPA (n = 7), 6-OHDA saline (n = 9), 6-OHDA L-DOPA (n = 10) and 6-OHDA L-DOPA+STN lesion (n = 8). R = right, ipsilateral to sham or 6-OHDA injection; L = left, contralateral to sham or 6-OHDA injection. Data are expressed as mean ± S.E.M. *** *p*<0.001 (one-way ANOVA, followed by Bonferroni *post-hoc*).

Chronic L-DOPA administration (6 mg/kg plus benserazide 12 mg/kg) rapidly induced dyskinesia in 6-OHDA lesioned rats. This dosage has been extensively used to improve motor impairment and also to induce LID in this model of PD [20], [29]–[36]. During the first week of treatment AIMs increased, reaching a plateau by the second week (F_(7,112)_ = 38.03, *p*<0.001; Figure 2A). Locomotive AIMs increased over time, but showed large variability between animals and testing sessions (F_(7,112)_ = 4.07, *p*<0.001; Figure 2B). As expected, we did not observe AIMs in any of the control groups, including sham animals treated with saline or L-DOPA, and 6-OHDA lesioned rats treated with saline. Evaluation of the time course (180 min) of the AIMs after a single injection of L-DOPA showed the expected profile [20], [36] ((F_(7,112)_ = 89.67 and F_(6,96)_ = 33.95, *p*<0.001; Figure 2C and D respectively) with the first signs appearing 10–20 min after the drug injection, reaching a peak between 40–80 min and subsiding by 140–180 min.

The extent of the nigral lesion and striatal DA denervation were confirmed later by TH immunohistochemistry for animals used in experiment 1 (n = 52; Figure 2E) and by western blot for experiments 2 and 3 (n = 34; Figure 2F).

### Electrophysiological characterization of the subthalamic nucleus in hemiparkinsonian rats with L-DOPA induced dyskinesia

**Figure 3:**
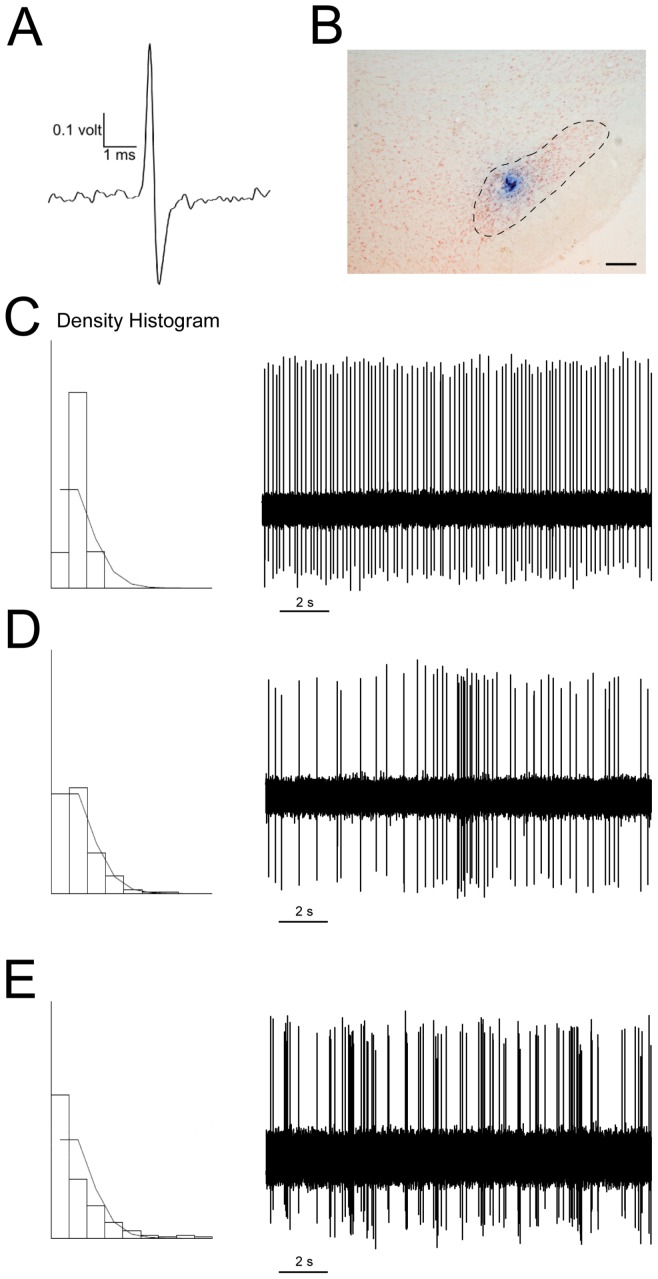
Electrophysiological characterization of neurons in the subthalamic nucleus. *(* ***A*** *)* A single spike from an STN neuron recorded *in vivo*. *(* ***B*** *)* Histological verification of the recording site in the STN, counterstained with neutral red. Scale bar: 200 µm. Examples of action potential traces, showing the three characteristic firing patterns of STN neurons. *(* ***C*** *)* Tonic firing pattern, in which the density histogram follows a Gaussian distribution. *(* ***D*** *)* Random firing pattern, in which the density histogram represents a Poisson distribution. *(* ***E*** *)* Bursting firing pattern, in which the density histogram represents a distribution significantly different from a Poisson distribution, with a significantly positive skewness of the density discharge distribution histogram.

**Figure 4:**
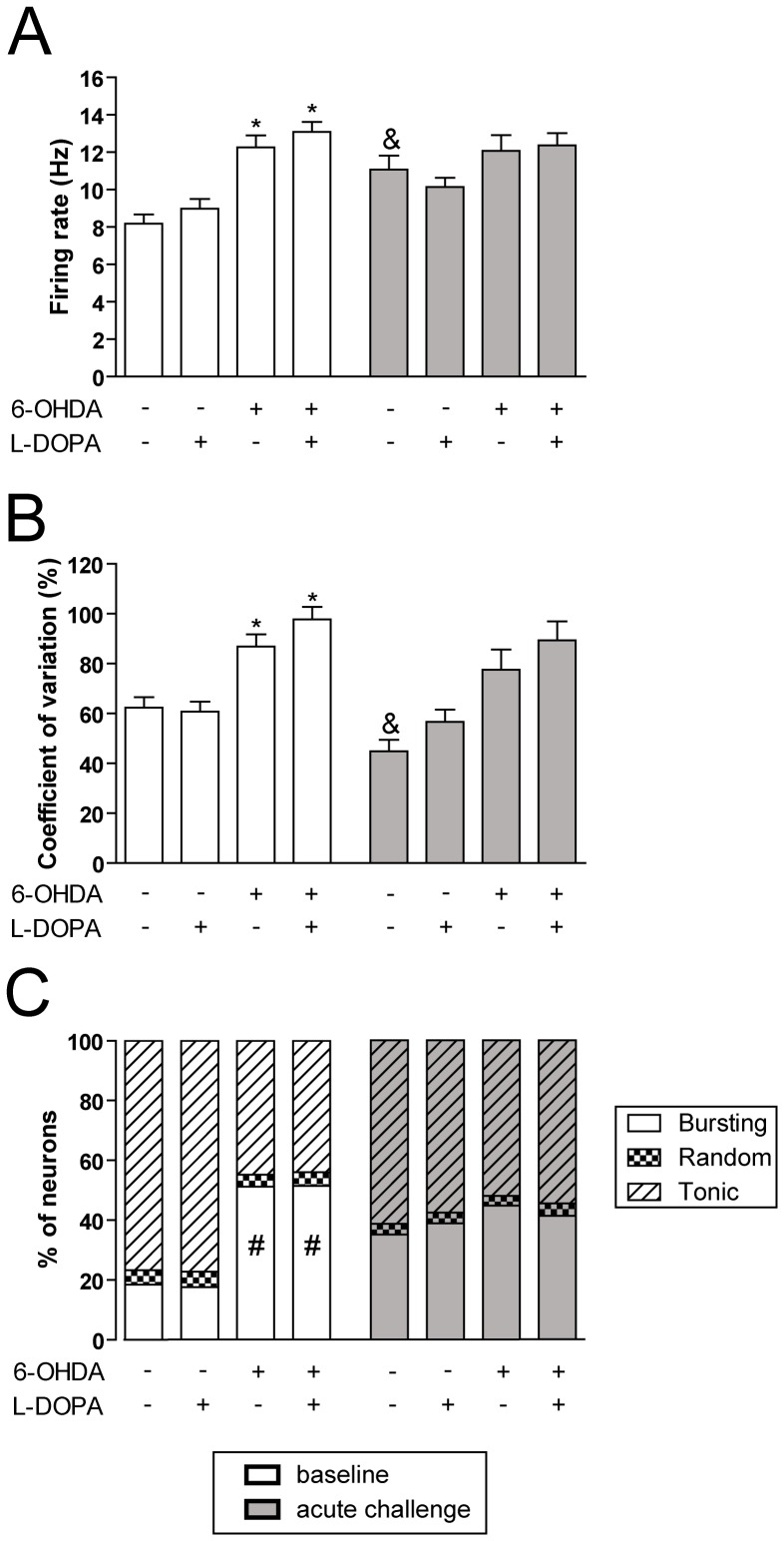
Electrophysiological characterization of the subthalamic nucleus. *(* ***A*** *)* Firing rate of STN glutamatergic neurons. Baseline firing rate (white bars) was significantly different between the groups (F_(4,307)_ = 15.25, *p*<0.0001, one-way ANOVA). The acute L-DOPA challenge (grey bars), induced an increase in firing rate in sham saline animals only. *(* ***B*** *)* Coefficient of variation (%). Baseline activity recording (white bars) showed significant differences between groups (F_(4,307)_ = 11.85, *p*<0.0001, one-way ANOVA) and acute L-DOPA treatment produced a significant reduction in the sham saline group. *(* ***C*** *)* Percentage of bursting, random and tonic neurons. Baseline burst firing pattern (white bars) was significantly increased in 6-OHDA lesioned groups (*p*<0.05, Fisher's test). Both sham groups showed a trend to increase the number of bursting neurons after an acute L-DOPA dose (sham saline, *p* = 0.08, and sham L-DOPA, *p* = 0.06 vs its baseline). Data are expressed as mean ± S.E.M. All data included in the acute L-DOPA challenge were obtained between 20 and 120 min after L-DOPA injection (time period corresponding to high dyskinetic behaviour). * *p*<0.05 vs sham saline and sham L-DOPA (one-way ANOVA, followed by Bonferroni *post-hoc*). ^&^ *p*<0.05 vs before L-DOPA administration (unpaired t test). ^#^ *p*<0.05 vs sham saline and sham L-DOPA (Fisher's exact test for firing pattern).

To investigate the effect of L-DOPA chronic treatment on the STN activity, neurons from sham and 6-OHDA lesioned rats treated with saline or L-DOPA were recorded 24 h after the last saline or L-DOPA dose (baseline). All cells recorded showed a biphasic action potential waveform with a duration of 1.0–1.5 ms (Figure 3A), were localized within the STN (Figure 3B) and displayed the characteristic firing patterns of STN neurons (Figure 3C, 3D and 3E). A total of 309 glutamatergic neurons were recorded in the STN: 51 neurons from sham saline group, 55 neurons from sham L-DOPA group, 60 neurons from 6-OHDA saline group and, 143 neurons from 6-OHDA L-DOPA group (Figure 4). We found significant differences between the sham and the 6-OHDA lesioned groups in the baseline firing rate (Figure 4A), regularity (Figure 4B), and pattern (Figure 4C), consistent with hyperactive STN features typical of DA denervated states. The mean firing rate was higher in the 6-OHDA saline animals compared with the sham saline group (12.26±0.64 Hz vs 8.18±0.49 Hz, *p*<0.05), and in the 6-OHDA L-DOPA group compared with sham L-DOPA (13.09±0.53 Hz vs 8.97±0.52 Hz, *p*<0.05). In the 6-OHDA saline group the firing activity was more irregular (the variation coefficient was 86.88±4.88% vs 62.33±4.20%, *p*<0.05) and the bursting activity was higher (51.22% vs 18.43%, *p*<0.05) and the amount of neurons showing a tonic (44.76% vs 76.72%) pattern was lower than that found in neurons recorded from the sham saline group, while the number of neurons showing a random (4.02% vs 4.85%) pattern in both groups was not different. Comparison between electrophysiological parameters obtained from 6-OHDA saline and 6-OHDA L-DOPA groups did not reveal any difference indicating that chronic treatment with L-DOPA did not modify the STN hyperactivity induced by 6-OHDA lesion.

In order to investigate the sensitivity of the STN neurons to L-DOPA treatment, electrophysiological recordings were also obtained from the same rats 20–120 min after an acute challenge of L-DOPA (this time period corresponds to intense dyskinetic behaviour). A total of 149 glutamatergic neurons were recorded (Figure 4). In the acute L-DOPA challenge experiment (Figure 4A, 4B and 4C, grey bar histograms) we only detected significant differences from baseline in the sham saline group. In this group STN neurons responded to the L-DOPA challenge with a significant increase in the firing rate with respect to baseline (11.07±0.74 Hz vs 8.18±0.49 Hz, *p*<0.05; Figure 4A), and a significant reduction in the coefficient of variation (44.88±4.57% vs 62.33±4.20%, *p*<0.05; Figure 4B). The number of bursting neurons tended to increase both in sham saline and sham L-DOPA groups (Figure 4C). We did not find any other significant effects of acute L-DOPA in the rest of the experimental groups.

We also investigated whether there was a correlation between the AIMs score in the last testing session (day 21^st^) and STN electrophysiological parameters (firing rates and coefficient of variation) as summarised in Table 1. Although no correlation was observed for the basic AIM scores, we observed a significant correlation between the axial global AIM score and the baseline firing rate that was not longer present after the acute L-DOPA challenge.

**Table 1:** Regression analysis between scored global AIMs and STN neurons electrophysiological parameters in dyskinetic animals.

Integrated AIMs score	Before L-DOPA		After L-DOPA	
Abnormal Involutary Movements	Firing rate	Coefficient of variation	Firing rate	Coefficient of variation
**Ax+Li+Or**	R = 0.36	R = −0.13	R = 0.24	R = 0.31
	ns	ns	ns	ns
**Axial**	R = 0.46*	R = −0.03	R = 0.15	R = 0.35
	P = 0.036	ns	ns	ns
**Limb**	R = 0.27	R = −0.28	R = 0.25	R = 0.26
	ns	ns	ns	ns
**Orolingual**	R = 0.03	R = −0.19	R = 0.41	R = 0.10
	ns	ns	ns	ns
**Locomotive**	R = 0.21	R = −0.31	R = 0.18	R = 0.24
	ns	ns	ns	ns

Pearson r values for correlation between the global AIMs and the electrophysiological parameters of STN neurons recorded 24 hours after the last L-DOPA injection, and neurons recorded from 20 to 120 minutes after L-DOPA administration,

* *p*<0.05.

In summary, these results show that chronic L-DOPA treatment did not modify the abnormal STN activity induced by the 6-OHDA lesion and that there was no correlation between AIM scores and electrophysiological parameters, except for the axial AIM subscore.

### Effect of subthalamic nucleus lesion on L-DOPA-induced dyskinesia

**Figure 5:**
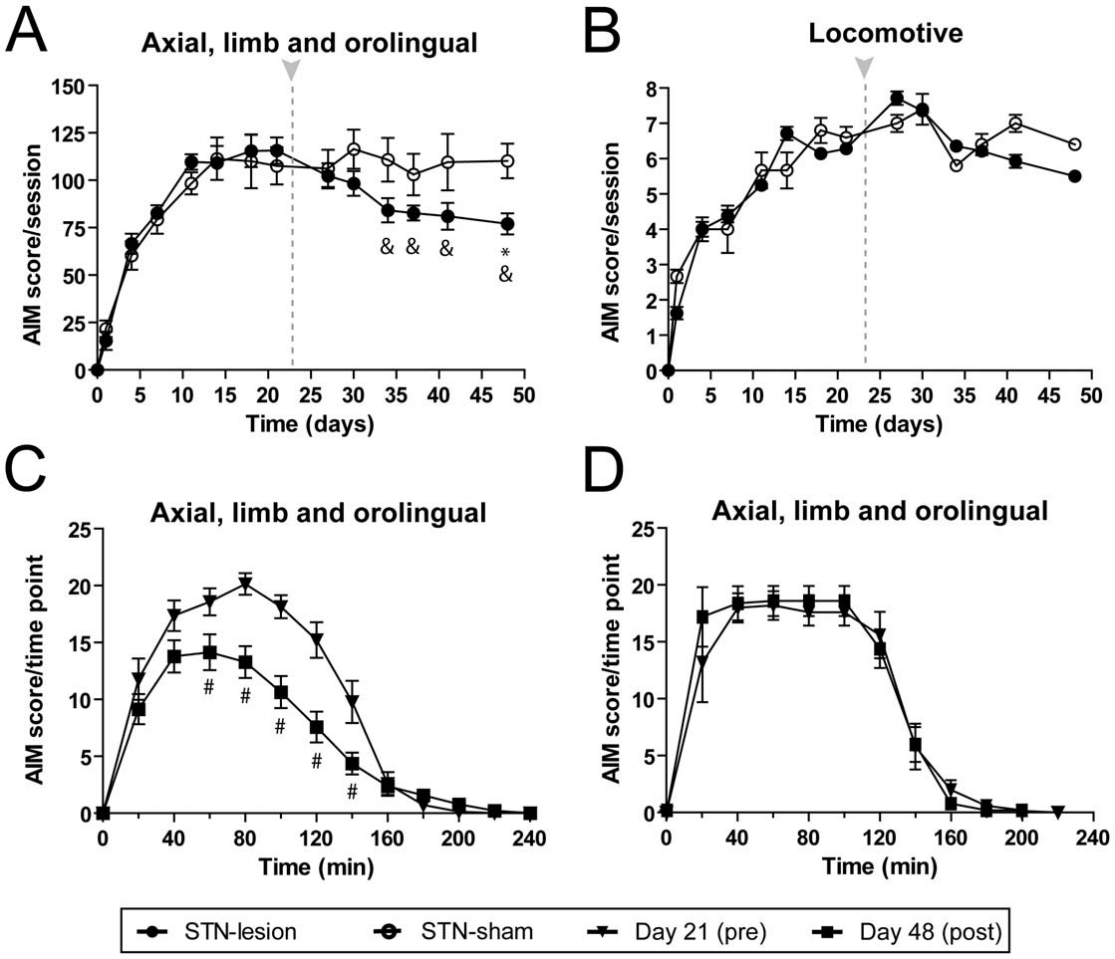
Effect of subthalamic nucleus lesion on the severity of L-DOPA-induced dyskinesia. Evolution of *(* ***A*** *)* AIM scores/session, showing significant reduction after STN lesion in the sum of axial, limb and orolingual AIMs scored per session, whereas *(* ***B*** *)* locomotive AIMs scored per session were similar in both groups. The arrow marks the time of STN injection at day 22. Graphical representation of AIM scores/time point (AUC) comparing the day before STN injection (day 21^st^, black triangle) and the last testing session after STN injection (day 48^th^, black square). Time course of axial, limb and orolingual AIMs scored in *(* ***C*** *)* STN-lesion and *(* ***D*** *)* STN-sham animal groups. The results show a significant decrease in AIMs scores after STN lesion. Groups: 6-OHDA L-DOPA+STN-lesion (n = 14) and 6-OHDA L-DOPA+STN-sham (n = 5). Data are expressed as mean ± S.E.M. ^&^ *p*<0.05 vs day 21st (one-way RM ANOVA, followed by Bonferroni post-hoc). * *p*<0.05 vs STN-sham (two-way RM ANOVA, followed by Bonferroni *post-hoc*). ^#^ *p*<0.05 (two-way RM ANOVA, followed by Bonferroni *post-hoc*).

In order to clarify the involvement of the STN in LID, a group of dyskinetic rats were injected with ibotenic acid (STN-lesion group) or vehicle (STN-sham group) into the STN ipsilateral to the 6-OHDA lesion. Five days later, AIM rating was continued for 6 additional sessions. Before the STN injection there were no differences in AIM scores between groups (Figure 5A). After the injection, animals in the STN-lesion group showed a reduction in the AIM scores compared to their pre-lesion scores (F_(6,78)_ = 7.418, *p*<0.001; Figure 5A) while in the STN-sham group there were no changes between pre- and post-injection scores. Post injection scores were significantly different between the two groups (F_(1,85)_ = 5.513, *p*<0.05; Figure 5A). On the other hand, locomotive AIM scores were not modified by the STN lesion (Figure 5B and data not shown).

Analysis of the time course (area under the curve, AUC) of the AIMs pre- (day 21^st^) and post- (day 48^th^) STN injection showed a significant reduction in the the STN-lesion group (F_(1,286)_ = 17.05, *p*<0.001; Figure 3C), while the AUC was not modified by the sham injection (Figure 5D).

**Figure 6:**
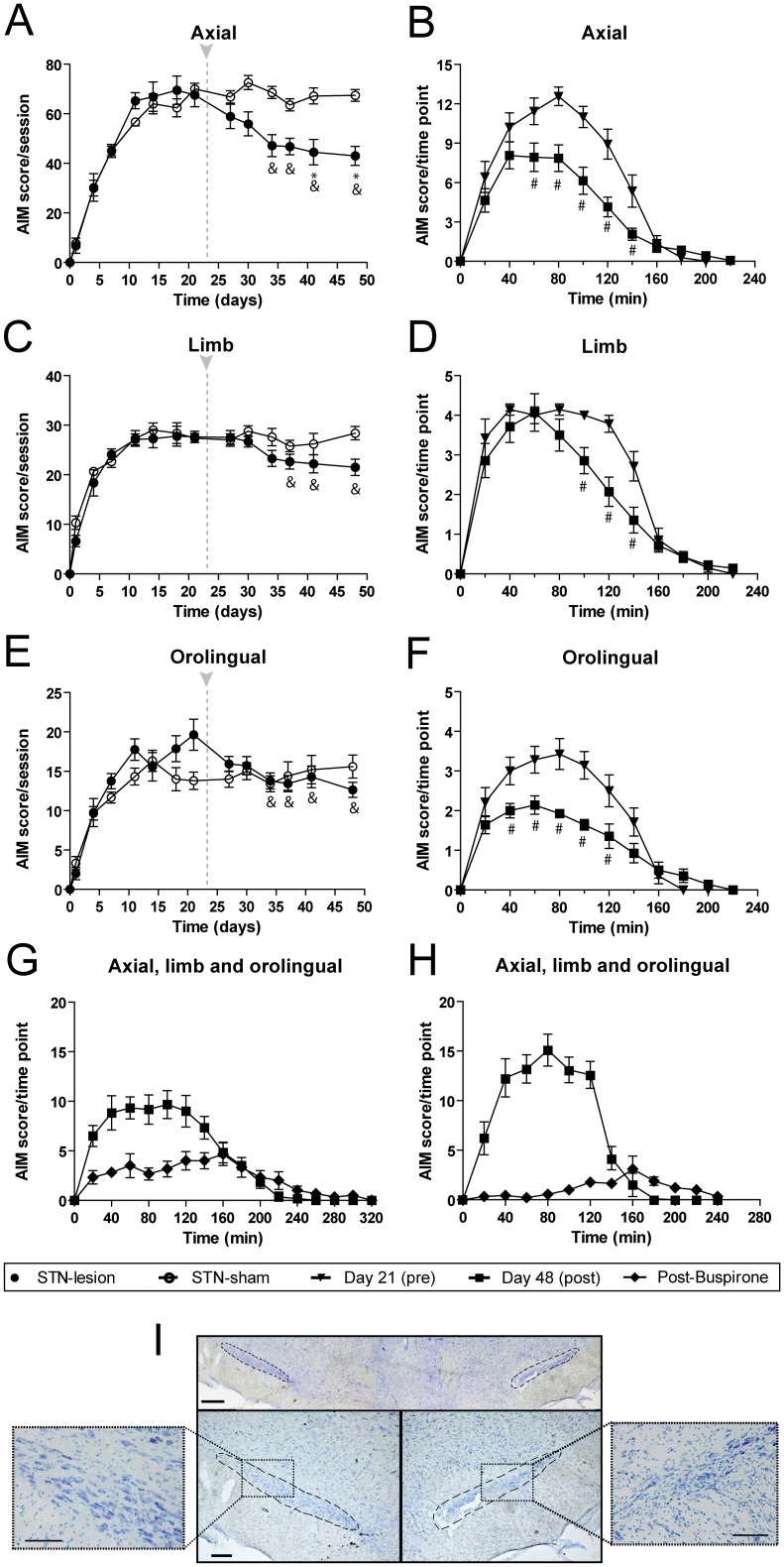
Effect of subthalamic nucleus lesion on the severity of the different subtypes of L-DOPA-induced dyskinesia. Evolution of AIM scores/session of the *(* ***A*** *)* axial, *(* ***C*** *)* limb and *(* ***E*** *)* orolingual AIMs pre- and post-STN injection, comparing STN-lesion and STN-sham groups. Time course of score for each subtype of AIM pre- and post-STN injection; there were significant differences between pre- and post- injection in the STN-lesion in *(* ***B*** *)* axial, *(* ***D*** *)* limb and *(* ***F*** *)* oral subtypes. Groups: 6-OHDA L-DOPA+STN-lesion (n = 14) and 6-OHDA L-DOPA+STN-sham (n = 5). *(* ***G*** *)* The response to buspirone was maintained after the STN lesion, but the effect was significantly smaller than in the *(* ***H*** *)* STN-sham group. Groups: 6-OHDA L-DOPA+STN-lesion (n = 6) and 6-OHDA L-DOPA+STN-sham (n = 5). *(* ***I*** *)* Thionine-staining of the STN (upper panel). The bottom panels correspond to higher magnification of the ipsilateral (right) and contralateral (left) sides. Scale bar: 400 µm (upper panel) and 200 µm (lower panel). Data are expressed as mean ± S.E.M. ^&^ *p*<0.05 vs day 21st (one-way RM ANOVA, followed by Bonferroni). * *p*<0.05 vs STN-sham (two-way RM ANOVA, followed by Bonferroni *post-hoc*). ^#^ *p*<0.05 (two-way RM ANOVA, followed by Bonferroni *post-hoc*).

We analyzed separately the effect of the STN lesion on the scores for axial, limb and orolingual AIMs subtypes. After the STN lesion, the axial AIM scores were significantly reduced (F_(6,78)_ = 6.481, *p*<0.001; Figure 6A) and significantly different from post-injection scores in the STN-sham group (F_(1,85)_ = 8.508, p<0.01; Figure 6A). Additionally, the axial scores during the last testing session were also reduced after the lesion (F_(1,286)_ = 15.95, *p*<0.001; Figure 6B). Limb AIM scores showed a similar profile and were significantly attenuated by the lesion (F_(6,78)_ = 6.941, *p*<0.001; Figure 6C and F_(1,286)_ = 7.986, *p*<0.01, Figure 6D). Likewise, orolingual AIM scores were significantly reduced (F_(6,78)_ = 4.236, *p*<0.01; Figure 6E and F_(1,286)_ = 10.25, *p*<0.01; Figure 6F). None of the AIMs subtypes was different after sham injection in the STN (data non shown).

It has been shown that buspirone effectively decreases LID [37]. Thus, in order to better understand the effect of STN ablation, we next tested the effect of buspirone administered 30 min before L-DOPA. Buspirone significantly decreased the AIM scores in both groups, but the effect was blunted in the STN-lesion group (Figure 6G). AIM scores decreased by 83% in the STN-sham and by 47% in the STN-lesion group (*p*<0.01) (Figure 6H and 6G, respectively). Importantly, the difference in the effect of buspirone was roughly equivalent to the effect of the STN lesion (−32%), suggesting partially overlapping mechanisms.

At the end of the experiment, the STN lesion was verified by thionine staining (Figure 6I). Microscopic analysis showed loss of large STN neurons and quantification of STN cell numbers by stereological methods showed a reduction of 62.2±2.6% with respect to the intact side.

### Effect of subthalamic nucleus lesion on striatal protein expression

To evaluate the impact of the STN lesion on striatal proteins involved in dyskinesia, animals were killed one hour after the last saline or L-DOPA treatment and the striata were removed for protein analysis.

**Figure 7:**
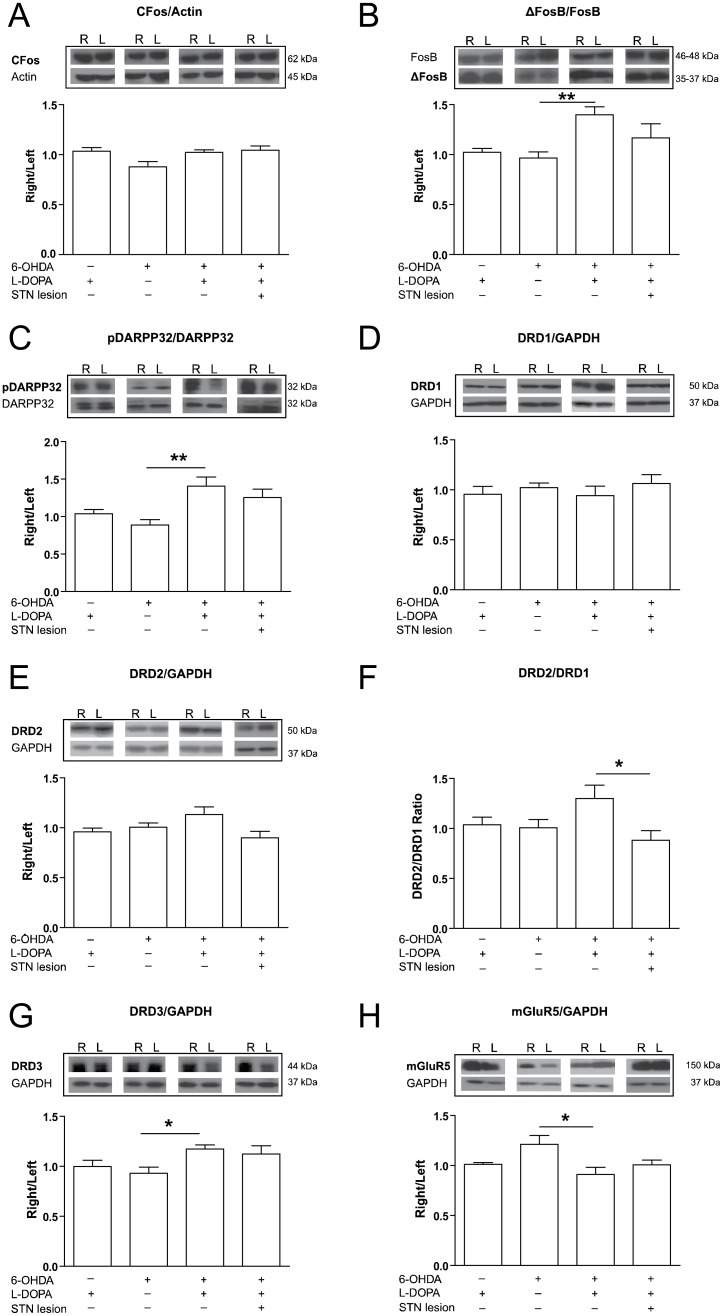
Effect of subthalamic nucleus lesion in protein expression in the striatum. *(* ***A*** *)* Western blot representative images (upper panel) and quantification (lower panel) of cFos protein showing no significant differences between the groups. *(* ***B*** *)* Quantification of ΔFosB/FosB western blot showed significant increased in 6-OHDA L-DOPA group versus 6-OHDA saline group ratio, indicating that L-DOPA treatment increase ΔFosB expression in the lesioned striatum. *(* ***C*** *)* pDARPP32/DARPP ratio was significantly higher in lesioned animal treated chronically with L-DOPA. *(* ***D*** *)* Results of the analysis of DRD1 and *(* ***E*** *)* DRD2 expression showing no significant differences between the groups. *(* ***F*** *)* Analysis of DRD2/DRD1 ratio showed a significant increase in 6-OHDA L-DOPA group. *(* ***G*** *)* DRD3 was significantly higher in 6-OHDA treated with L-DOPA compared to the animals that received saline. *(* ***H*** *)* mGluR5 protein expression quantification revealed a significant reduction in 6-OHDA L-DOPA group versus 6-OHDA saline group. Groups: sham L-DOPA (n = 7), 6-OHDA saline (n = 9), 6-OHDA L-DOPA (n = 10) and 6-OHDA L-DOPA+STN-lesion (n = 8). R = right, ipsilateral to sham or 6-OHDA injection; L = left, contralateral to sham or 6-OHDA injection. Data are expressed as mean ± S.E.M. * *p*<0.05 and ** *p*<0.01 (one-way ANOVA, followed by Bonferroni *post-hoc*).

First, we examined the expression of cFos protein that belongs to the immediate early gene family of transcription factors. Analysis of the western blots did not show significant differences in the expression of this indirect marker of neuronal activity (Figure 7A). We then analyzed the expression of ΔFosB, a stable truncated splice variant of FosB that has been associated with L-DOPA induced dyskinesia [38]. ΔFosB/FosB ratio was significantly different between the groups (F_(3,30)_ = 4.884, *p*<0.01) and the post hoc analysis showed that the ratio was significantly higher in L-DOPA treated animals in comparison with saline treated animals (1.39±0.11 vs 0.96±0.006; Figure 7B). Levels in the STN-lesion group fell in between (1.16±0.14) and were not significantly different from either saline or L-DOPA groups.

Next, we examined phosphorylated DARPP-32 (dopamine and adenosine 3′ 5′ monophospate-regulated phosphoprotein-32K) in Thr34, the inhibitory catalytic subunit of protein phosphatase 1 (PP1). We found that its expression was significantly different between the groups (F_(3,30)_ = 5.406, *p*<0.01; Figure 7C), with a significant increase in the 6-OHDA L-DOPA animals compared with the 6-OHDA saline animals (1.41±0.11 vs 0.89±0.07). The STN-lesion group showed intemediate levels (1.25±0.11), which were not significantly different from the other groups.

We then determined the expression of DA receptors, DRD1, DRD2 and DRD3 in the striatum. The results showed that there were no significant differences between groups in the expression of either DRD1 (Figure 7D) or DRD2 (Figure 7E). However, we found that the DRD2/DRD1 ratio showed significant differences between the groups (F_(3,30)_ = 3.359, *p*<0.05). Indeed, there was a consistent trend for DRD2 to increase and DRD1 to decrease in the L-DOPA treated dyskinetic animals, (DRD2/DRD1 ratio 1.29±0.13) and this ratio was significantly higher than in the STN-lesion group (0.88±0.09). There were also significant differences between the groups in the expression of DRD3 (F_(3,30)_ = 3.295, *p*<0.05; Figure 7G), that was significantly higher in the L-DOPA than in the 6-OHDA saline group (1.17±0.04 vs 0.93±0.05). The STN-lesion group showed intermediate levels of DRD3 (1.12±0.09) that were not significantly different from either the control or the dyskinetic groups. Thus, we found that while the STN lesion did not induce a significant effect on any of the markers examined, the aberrant pattern characteristic of LID was attenuated.

Finally, we examined the expression of mGluR5, which has been implicated in L-DOPA induced dyskinesia and could be altered by the STN lesion. The results showed that the expression pattern was significantly different between the groups (F_(3,30)_ = 3.821, *p*<0.05), and the post hoc analysis revealed that mGluR5 expression was higher in the group of 6-OHDA saline than in the L-DOPA treated animals (1.21±0.08 vs. 0.91±0.07; Figure 7H). These results suggest that the 6-OHDA lesion up-regulates mGluR5 expression in the denervated striatum, whereas chronic L-DOPA administration appears to normalize its expression in all the groups.

## Discussion

In this study we examined the role of STN in LID in a well-characterized hemiparkinsonian rat model [36]. STN lesion, or deep brain stimulation (DBS) using high frequency stimulation (HFS), efficiently reverse motor symptoms in PD patients [39]–[42] and in experimental models of nigrostriatal DA deficiency [43], [44]. However, the impact of STN manipulation on LID remains poorly defined. The results of this study show that the STN has a role, if modest, in the physiopathology of dyskinesias. Ours is the first report to address in a systematic way the impact of STN lesion on the behavioral and molecular aspects of dyskinesia.

### STN activity and Abnormal Involuntary Movements

We found that the DA denervation increased STN activity, as predicted by the classical basal ganglia model [6] and previously shown by us and others [8]–[11], [45], [46]. Acute L-DOPA administration increased the firing activity of STN neurons in sham saline group. In addition, L-DOPA treatment did not modify the STN hyperactivity and the acute effect of L-DOPA on STN was abolished after nigrostriatal degeneration and was not recovered by prolonged L-DOPA treatment. These findings suggest a desensitization of the DA receptors that modulate STN activity [47]–[50]. Alternatively, the lack of effect of L-DOPA on the STN activity may be due to the loss of the SN input [51]–[53] after 6-OHDA lesion which may reduce L-DOPA conversion into DA. Indeed, microiontophoretic administration of DA exerts a direct action on STN neurons [54]. It could be argued that the lack of L-DOPA effect on STN activity was due to the low dosage selected for the study. Indeed, an acute administration of 100 mg/kg of L-DOPA (i.v.) to 6-OHDA lesioned rats has been described to reduce the augmented firing rate of STN neurons [55]. However, the changes observed on STN activity after chronic L-DOPA treatment are not uniform. Thus, both normalization [56] and no effect have been reported on MPTP treated monkeys [57] and in patients with dyskinesias induced by apomorphine [58] with higher doses than the one used in the present study. Although we cannot exclude the fact that this low dose (6 mg/kg) of L-DOPA was insufficient for reversing the abnormal firing rate and pattern of STN neurons observed in 6-OHDA lesioned rats, we consider that this is unlikely as it was sufficient to induce molecular and behavioral changes and to modify the firing rate in control animals. On the other hand, we observed a positive correlation between the baseline firing rate and the axial global AIM in the 6-OHDA L-DOPA group.

### STN chemical ablation attenuates behavioral and molecular changes associated with LID

An important finding in this study is the behavioral improvement following STN lesion. Notwithstanding, the reduction was modest (∼30%) and more pronounced when comparing pre and post-STN lesion peak-dose scores, indicating that the subthalamotomy reduced the severity but not the presence and duration of LID. At the molecular level the striatal profile after STN ablation was not significantly different from the dyskinetic group, but neither from the non-dyskinetic controls, thus showing a parallel normalization of the aberrant changes associated with LID and striatal plasticity.

Although many studies point to the DRD1- direct pathway as responsible for the development of LID [59], there is growing consensus for the importance of a balanced striatal outflow. In this study we did not find significant changes in DRD1 or DRD2 protein expression but the ratio DRD2/DRD1 was significantly increased, supporting the hypothesis that the loss of balance between DA inhibitory and facilitating pathways underlies striatal aberrant plasticity [60] and LID. In this context, the involvement of DRD3 is complex as it can couple to Gs and Gi proteins in downstream activation of signaling pathways. We previously described, using functional magnetic resonance imaging, a DRD1-like response (increase in cerebral blood volume) in response to a DRD3 agonist in dyskinetic rats and primates [61] suggesting that sensitized DRD3 receptors are able to activate DRD1 transduction pathways. Our results here show that in spite of a decrease in DRD1 relative to DRD2 in dyskinetic animals, the levels of pDARPP32, phosphorylated in Thr34 are elevated, suggesting that the DRD1 canonical pathway (Santini et al., 2010) could perhaps be activated downstream DRD3. Interestingly DRD3 antagonists have a potent dyskinetic effect [62], [63].

We confirmed an up-regulation of mGluR5 in response to DA denervation, which we have previously observed *in vivo* in parkinsonian primates using PET [64]. Several studies have demonstrated that mGluR5 receptor antagonists have antiparkinsonian effects in 6-OHDA treated rats [65], and reduce the toxicity of MPTP in mice [66]–[69]. A few studies have also reported antidyskinetic effects of mGluR5 antagonists [70]–[75]. In our study it was somewhat unexpected to find a normalization of the mGluR5 ratio in the dyskinetic group, which was secondary to a small increase in the contralateral (unlesioned) side in animals receiving L-DOPA.

### STN role in dyskinesia: possible mechanisms

The SN receives a direct excitatory input from the STN [76], [77]. STN lesion or HFS-related decrease in glutamate excitatory drive on the intact SNc (SN *pars compacta*) results in a decrease in SNc activity and DA release in the striatum [78]. In the parkinsonian state, the STN is hyperactive and thus removal of the glutamate input (that could now be excitotoxic) from STN to the SNc can protect the remaining DA cells - as demonstrated in primates, even when the STN lesion (or HFS) takes place after the DA lesion [79]. In contrast with the effect in intact animals, STN ablation (or HFS) in parkinsonian animals is associated with increased striatal DA levels and prolongation of the effect of L-DOPA [80], [81]. In this study we did not find significant differences between the groups in the extent of DA denervation, but the pattern of molecular changes was consistent with an increase in DA tone in the striatum, with a tendency to normalize dyskinesia related changes, albeit incompletely. Indeed, a functional positron emission tomography (PET) study using raclopride (a DRD2 low affinity agonist) displacement to measure DA release in patients with STN HFS, found a significant attenuation of L-DOPA induced fluctuations in striatal DA levels [82]. However, other studies reported no evidence of increase striatal DA release during STN HFS [83]–[85], so this hypothesis needs to be confirmed.

The fact that the effect of subthalamotomy was not very intense (about 30%) suggests that the STN does not have a determinant role in the mechanism underlying dyskinesias. Nonetheless, the concomitant blunted response to the potent anti-dyskinetogenic effect of buspirone [86] after the STN ablation, ratifies the robustness of this contribution. These results also suggest that buspirone acts partly through modulation of the indirect pathway given that interruption of this loop by the STN lesion significantly weakened its effect. Buspirone effect on dyskinesia has been attributed to its partial agonist effect on 5HT1A receptors [37], [87]. Interestingly, a recent communication pointed to an effect of buspirone through blockade of hypersensitive DRD2 receptors on graft-induced dyskinesia [88], thus the improvement after STN lesion could be due to a normalization of DA receptor sensitivity. Importantly, in PD patients with STN DBS the improvement in dyskinesias (∼70%) is larger than the reduction in L-DOPA dose (35–40%) [89], [90] -unlike for other complications, like motor fluctuations. Nevertheless, it has been difficult to demonstrate a role of STN cancellation in dyskinesia beyond the reduction in daily L-DOPA dose in patients. Taking together the behavioral, electrophysiological and molecular data our study shows that the STN is involved in the modulation of the dyskinetogenic effects of L-DOPA therapy and suggests an indirect effect, perhaps by increasing striatal DA tone that awaits future confirmation.
